# Clinical Research on the Mechanisms Underlying Immune Checkpoints and Tumor Metastasis

**DOI:** 10.3389/fonc.2021.693321

**Published:** 2021-07-22

**Authors:** Xi-Yang Tang, An-Ping Shi, Yan-Lu Xiong, Kai-Fu Zheng, Yu-Jian Liu, Xian-Gui Shi, Tao Jiang, Jin-Bo Zhao

**Affiliations:** ^1^ Department of Thoracic Surgery, Tangdu Hospital, Air Force Medical University, Xi’an, China; ^2^ Department of Radiology & Functional and Molecular Imaging Key Lab of Shaanxi Province, Tangdu Hospital, Fourth Military Medical University (Air Force Medical University), Xi’an, China; ^3^ College of Basic Medicine, Air Force Medical University, Xi’an, China

**Keywords:** immune checkpoint, immune therapy, immune response, tumor, metastasis

## Abstract

This study highlights aspects of the latest clinical research conducted on the relationship between immune checkpoints and tumor metastasis. The overview of each immune checkpoint is divided into the following three sections: 1) structure and expression; 2) immune mechanism related to tumor metastasis; and 3) clinical research related to tumor metastasis. This review expands on the immunological mechanisms of 17 immune checkpoints, including TIM-3, CD47, and OX-40L, that mediate tumor metastasis; evidence shows that most of these immune checkpoints are expressed on the surface of T cells, which mainly exert immunomodulatory effects. Additionally, we have summarized the roles of these immune checkpoints in the diagnosis and treatment of metastatic tumors, as these checkpoints are considered common predictors of metastasis in various cancers such as prostate cancer, non-Hodgkin lymphoma, and melanoma. Moreover, certain immune checkpoints can be used in synergy with PD-1 and CTLA-4, along with the implementation of combination therapies such as LIGHT-VTR and anti-PD-1 antibodies. Presently, most monoclonal antibodies generated against immune checkpoints are under investigation as part of ongoing preclinical or clinical trials conducted to evaluate their efficacy and safety to establish a better combination treatment strategy; however, no significant progress has been made regarding monoclonal antibody targeting of CD28, VISTA, or VTCN1. The application of immune checkpoint inhibitors in early stage tumors to prevent tumor metastasis warrants further evidence; the immune-related adverse events should be considered before combination therapy. This review aims to elucidate the mechanisms of immune checkpoint and the clinical progress on their use in metastatic tumors reported over the last 5 years, which may provide insights into the development of novel therapeutic strategies that will assist with the utilization of various immune checkpoint inhibitors.

## Introduction

**Figure 1:**
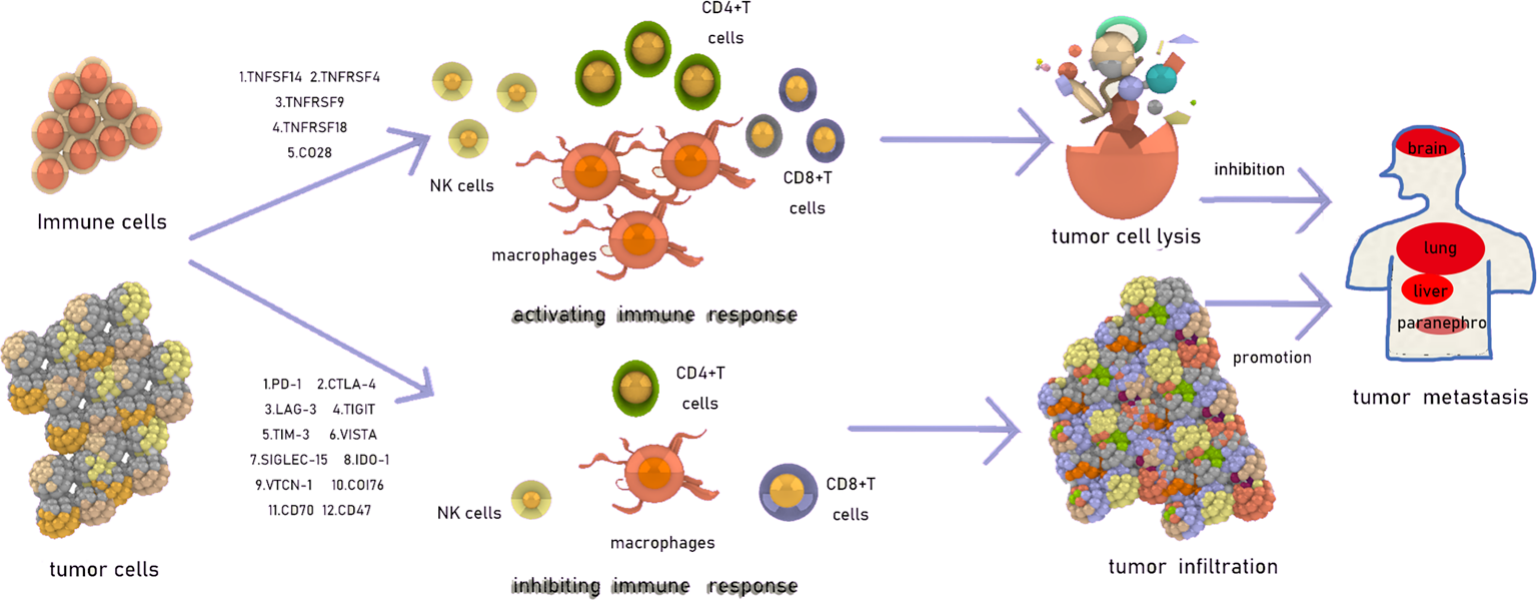
The introduction of immune checkpoints.

Avoidance of the surveillance of the immune system, through which tumor cell proliferation and growth can be successfully achieved, is one of the mechanisms of metastasis (1). The immune checkpoint is a type of receptor expressed on the surface of immune cells or tumor cells (2) that can negatively or positively regulate immune responses, in addition to mitigating immune injuries and preventing the development of autoimmunity, which may also be associated with tumor metastasis (3). In recent years, several studies have explored the curative effect of immune checkpoints in tumors and have subsequently investigated the underlying mechanisms of tumor metastasis (**Figure 1** **)**. The present study focused on 17 immune checkpoints, including those that exert negative effects, as programmed cell death 1 (PD-1), cytotoxic T lymphocyte associated protein 4 (CTLA-4), lymphocyte activating 3 (LAG-3), T cell immunoreceptor with Ig and ITIM domains (TIGIT), T cell immunoglobulin (Ig), and mucin domain-containing protein 3 (TIM-3), V domain-containing immunoglobulin suppressor of T-cell activation (VISTA, B7-H5), V-set domain containing T cell activation inhibitor 1 (VTCN1, B7-H4), CD276 (B7-H3), sialic acid binding Ig-like lectin 15 (SIGLEC-15), indoleamine 2,3-dioxygenase 1 (IDO1), CD70, and CD47, and positive effects, as TNF super family (TNFSF)14, TNF receptor super family (TNFRSF)4, TNFRSF9, TNFRSF18, and CD28, on the immune system. The types of immune-activating checkpoints are complex, while immune-inhibiting checkpoints are mainly composed of TNFSF and TNFRSF members; Moreover, immune-activating checkpoints exert their function mainly by activating effector T cells, while the immune-inhibiting ones not only inhibit T cell activation, but also accelerate T cell exhaustion as well as Treg induction (4–8).

T cell receptor (TCR) signaling pathways play a critical role in T cell activation, proliferation, and survival. Ras serves as a modulator in activating the Ras–ERK1/2–AP-1 signaling pathway, which induces IL-2 transcription. In mTORC1/2, an mTOR complex, the activation of mTORC2 induces the phosphorylation of AKT, which may be involved in TSC2 phosphorylation and mTORC1 activation, resulting in the activation of T cells; however, AKT may also be associated with the expression of PD-1 ligand (PD-L)1, which may downregulate T cell activation (9). The PKCθ–IKK–NF-κB, and IP3–Ca^2+^–NFAT pathways are other two signaling pathways associated with T cell activation. Furthermore, the phosphorylation and dephosphorylation of TCR—signaling molecules, such as Syk and ZAP-70—and the ubiquitination and degradation of CD3ζ, PKCθ, ZAP-70, phospholipase C-γ1, and phosphoinositide 3-kinase negatively regulate TCR signaling pathways. The positive and negative regulation of TCR signaling pathways maintains a balanced T cell activation (10, 11). Moreover, there are two main intersections between TCR signaling pathways and immune checkpoint pathways. Both ERK and AKT are associated with kinetic thresholds that control T cell activation. This phenomenon can also be controlled by immune checkpoints, and thus, immune checkpoint inhibitors (ICIs) may decrease the kinetic thresholds and influence T cell responsiveness to antigens (12). Therefore, the activation of T cells by ICIs may enhance the antitumor immune response, but side effects of ICIs, called immune-related adverse events (irAEs), may occur due to an excessive immune response resulting from the imbalance between Tregs and T_H_17 cells as well as increased production of cytokines, as TNF, IFN-γ, and IL-2. How to balance antitumor immunity and irAEs should be the focus of future research (13).

**Figure 2:**
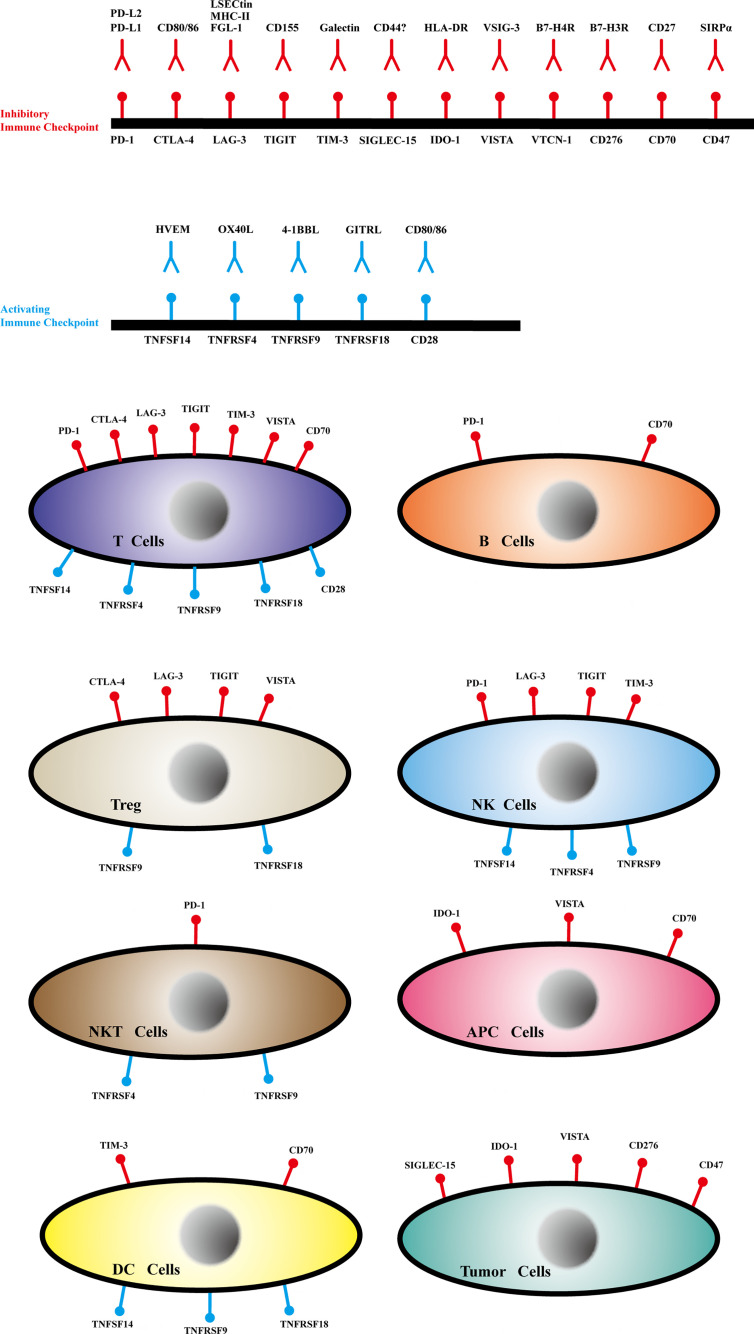
The ligands, receptors and expression of immune checkpoints.

Through this review, we aimed to highlight the latest research advances regarding immune checkpoint structure, expression (**Figure 2**), immune mechanisms (**Supplementary Figure S1**), relationship with tumor metastasis, and clinical applications to provide comprehensive information on immune checkpoints, which may aid the development of potential treatments for successful clinical therapy.

## Immune-Inhibiting Checkpoints

### PD-1 and PD-L1/2

#### Structure and Expression

##### The Immune Mechanism Related to Tumor Metastasis

PD-1 was first discovered in 1992 (14) and is a type I transmembrane protein with 288 amino acids and a single IgV extracellular domain (15). PD-1 is a receptor expressed on the surface of certain immune cells, such as activated T cells (ATCs), B cells, NKT cells, NK cells, and macrophages (15). PD-L1 and PD-L2 are the ligands of PD-1. PD-L1 is more widely distributed than PD-L2, being expressed on T cells, B cells, macrophages, and DC. In addition, PD-L1 is present on the surface of tumor cells, such as lung, colorectal (CRC), and breast cancers, as well as on the surface of melanoma cells, while PD-L2 is expressed restrictedly on macrophages and activated DCs (16).

PD-1 can inhibit the proximal TCR and CD28 (a type of T cell co-stimulatory receptor) and consequently attenuate ATC co-stimulation by inhibiting the activation of peripheral ATCs (17). Donnell et al. showed that the PI3K–AKT–mTOR axis promotes tumor proliferation, invasion, and metastasis (18). Tang et al. have concluded that the tumor microenvironment is influenced by PI3K–AKT, which is related to tumor metastasis (19). It is well established that mTOR is associated with T cell activation (10); however, hyperactivation of AKT was shown to promote PD-L1 expression in PTEN-mutated tumors. AKT as well as PD-1 and PD-L1 is upregulated, promoting their interaction on the surface of activated CD8^+^ T cells to induce T cell exhaustion and inhibition of antitumor immunity, which in turn further inhibits immunosurveillance, thereby leading to tumor growth and metastasis (9, 18). Thus, considering these findings, the development of various monoclonal antibodies (mAbs) directly targeting PD-1 or PD-L1 and research on PI3K–AKT–mTOR axis may help in achieving indirect inhibition of PD-1 or PD-L1. Furthermore, PD-L1 and PD-L2 downregulate tumor-specific T cell activation by inhibiting the cytotoxic T-cell immune response when binding to PD-1, which may be associated with tumor metastasis (20). Whether the expression of PD-1 can affect the tumor physical microenvironment and stromal immune cells or proteins in the extracellular matrix to influence the matrix mechanical or chemical traits warrants further investigation.

#### The Clinical Research Progress Related to Tumor Metastasis

PD-L1 may serve as a prognostic biomarker for tumor metastasis. Gao et al. have reported that the expression of PD-L1 increases significantly in metastatic prostate cancer and metastatic melanoma, indicating that the expression of PD-L1 may be relevant to the progression of these two cancer types (21). C5a blockade can be implemented in synergy with anti-PD-1 therapy to effectively activate CD8^+^ T cells, which in turn inhibit the proliferation and metastasis of lung cancer (22). Radiotherapy before anti-PD-1 treatment shows a favorable response rate for NSCLC patients; however, repeated radiotherapy may lead to increased PD-1 expression and NSCLC brain metastasis (23). Avelumab (MSB0010718c, anti-PD-L1 IgG1) is a novel mAb that is used to inhibit the interaction between PD-1 and PD-L1, and phase I and II clinical trials highlight that it is well tolerated and show its prognostic response in Merkel cell carcinoma (MCC), NSCLC, and urothelial carcinoma (24–27). The high expression of PD-1 is associated with metastatic breast cancer (MBC); however, a phase Ib trial has demonstrated unfavorable safety (incidence of grade ≥ 3 treatment-related AEs, 13.7%) and clinical effect (ORR, only 3% overall) of avelumab in the treatment of 168 patients with MBC (28). In a phase II clinical trial that was conducted to evaluate the sensitivity to anti-PD-1 therapy in metastatic triple-negative breast cancer, the performance of short-term cisplatin and doxorubicin treatment followed by nivolumab demonstrated significant improvements in patient outcomes; the ORR of cisplatin was 23% and that of doxorubicin was 35%, while the overall ORR was 20%, supporting that short-term cisplatin and doxorubicin treatment before nivolumab can improve the tumor microenvironment and enhance the sensitivity to anti-PD-1 therapy (29). In recurrent/metastatic nasopharyngeal carcinoma (RM-NPC), the safety of anti-PD-1 treatment with nivolumab and pembrolizumab was overall higher than that of camrelizumab, but the incidence of grade 3–5 AEs was also higher, which can be attributed to reactive capillary hemangiomas. In terms of effectiveness, the ORR of nivolumab, pembrolizumab, and camrelizumab was 19.0, 26.3, and 34.1%, respectively, indicating that camrelizumab demonstrated efficacy with relatively fewer AEs in patients with RM-NPC; however, there is no reasonable explanation for this difference in efficacy. Hence a question arises: Can camrelizumab be used in the treatment of other tumor metastases? Further exploration is warranted (30). Research on the response in patients with metastatic gastric cancer (mGC) has shown that EBV(+) and MSI-H are potential markers for pembrolizumab treatment in this type of cancer and can be used to predict the clinical response. Additionally, EP-32 can serve as an effective diagnostic indicator for liver metastasis of gastric cancer (31). In metastatic melanoma, PD-L1 expressed on many exosome surfaces is upregulated by IFN-γ, and concurrently, MAPK mediates cross-resistance in anti-PD-1 treatment (32, 33). Thus far, the Food and Drug Administration has approved the utilization of a considerable number of anti-PD-1 mAb such as nivolumab, pembrolizumab, and cemiplimab, and anti-PD-L1 mAb such as atezolizumab, avelumab, and durvalumab (34); however, no report has yet demonstrated that anti-PD-L2 mAb can be used clinically. Furthermore, several clinical trials that evaluate camrelizumab (anti-PD-1) efficacy and safety are underway. The data reviewed herein suggest the potential applicability of PD-1 or PD-L1, which provide a rationale for the use of anti-PD-1 or PD-L1 mAbs in the treatment of high-risk metastatic tumors.

### CTLA-4

#### Structure and Expression

##### The Immune Mechanism Related to Tumor Metastasis

CTLA-4 is inherently a membrane glycoprotein with a 36-amino acid cytoplasmic tail. CTLA-4 is a receptor mainly expressed on the surface of ATCs and Tregs.

CTLA-4 can inhibit the proliferation of self-reactive T cells rather than tumor-specific T cells by binding to B7 to competitively inhibit CD28 co-stimulation (35). Chen et al. have reported that CTLA-4 is not the main factor associated with tumor invasion and metastasis, owing to the above-mentioned mechanism and the lack of evidence, which may be attributed to the fact that the CTLA-4-specific mAb ipilimumab only demonstrates appreciable efficacy for metastatic melanoma and is less effective for other metastatic tumors (1). However, in a mouse model of colon cancer liver metastasis, anti-CTLA-4 therapy significantly increased the number of intra-tumoral CD8^+^ and CD4^+^ T cells, reducing the number of Tregs, inducing the production of various pro-inflammatory cytokines, such as IFN-γ, IL-1α, and IL-12, which are associated with CD8^+^ T cell activation and antitumor response, thereby effectively inhibiting liver metastasis of colon cancer (36). Similar findings have also been reported in a rectal cancer mouse model, in which anti-CTLA-4 therapy significantly inhibited liver metastasis accompanied by Treg suppression (37). Thus, CTLA-4 may play a role in tumor metastasis, especially that of CRC, by affecting immune cell function and cytokine secretion, but this role might not be significant in other tumors, as it may be masked by some unknown factors. Moreover, the immune mechanism underlying the occurrence of AE upon ipilimumab administration cannot be easily elucidated. Ipilimumab-associated irAEs include immune-related hepatitis, severe cutaneous reaction, and immune-related central nervous system symptoms such as headache, asthenia, dizziness, and balance disorder, which occur due to its basic immune mechanism. The subsequent and more extensive sections focus on the distinct mechanism underlying irAEs to prevent the occurrence of anti-CTLA-4 mAb-associated issues.

#### The Clinical Research Progress Related to Tumor Metastasis

It has been shown that combining an anti-CTLA-4 mAb (ipilimumab) with an anti-PD-L1 mAb (nivolumab) can help achieve satisfactory results in metastatic melanoma, metastatic CRC, and advanced renal cell carcinoma (34). Particularly, the double stimulation of anti-CTLA-4 and anti-PD-1 therapy to improve the immune system can help inhibit the proliferation and liver metastasis of colon cancer cells, and the progression-free survival (PFS) and median survival of patients increase by approximately 37% (36). Noteworthy, when an anti-CTLA-4 mAb is administered to patients with metastatic tumors, irAEs always occur. For example, the level of CTLA-4 always predicts the effect of ipilimumab therapy and the risk of developing irAEs in metastatic melanoma. Compared to patients presenting sCTLA-4 ≤200 pg/ml, those showing sCTLA-4 ≥200 pg/ml have a better response to ipilimumab and 40% less risk of death, but higher levels of CTLA-4 (≥200 pg/ml) are also associated with a higher incidence of irAEs, namely >5-fold increased risk of digestive tract irAEs, such as inflammatory bowel disease, ulcerative colitis, liver disease, and autoimmune pancreatitis (38). The latest research on metastatic tumor anti-CTLA-4 therapy shows a high risk of developing irAEs; the most remarkable irAEs include cutaneous diseases, like rash and pruritus, along with gastrointestinal tract irAEs, such as colitis. The most remarkable aspect highlighting the severity of these irAEs is that the incidence of all-grade irAEs is up to 72% and that of high-grade irAEs is up to 24%, suggesting that to achieve therapeutic benefits and to reduce irAEs attributable to anti-CTLA-4 administration, the above-mentioned aspects should be considered (39). The clinical data accumulated thus far provide unequivocal evidence that no effective approaches are available to inhibit the development of irAEs, and further studies are warranted to explore different combinations of anti-CTLA-4 mAbs and other mAbs to maximize their effect and minimize the AEs observed in the treatment of certain metastatic cancers. Owing to the fact that the causes of high-risk irAEs and their patterns of onset are unclear in anti-CTLA-4 treatment, in-depth studies of the potential molecular mechanisms are necessary in the early diagnosis and treatment of irAEs.

### LAG-3

#### Structure and Expression

LAG-3 consists of D1–D4, four immunoglobulin-like domains. There are three ligands of LAG-3 namely FGL-1, MHC-II, and LSECtin (40). FGL-1 is a member of the fibrinogen family, which is highly homologous with fibrinogen beta-and gamma-subunits carboxyl terminus (40). LAG-3 is mainly expressed on the surface of activated CD4^+^ T cells, CD8^+^ T cells, NK cells, and Treg cells (41).

#### The Immune Mechanism Related to Tumor Metastasis

Solinas et al. (42) have demonstrated that LAG-3 exerts a negative effect on the immune response *via* competitive binding to MHC-II; however, the intrinsic mechanism between LAG-3 and LSECtin (one of the several LAG-3 ligands) remains elusive. LAG-3 negatively regulates T cell proliferation accompanied by T cell exhaustion and can also lead to the activation of DC (43). The relationship between LAG-3 and MHC-II is universally accepted, demonstrating an affinity that is 40 times higher than that of CD4; however, the affinity to LSECtin and FGL-1 warrants further research.

#### The Clinical Research Progress Related to Tumor Metastasis

By analyzing the expression of LAG-3 on the surface of tumor-specific T cells, which inhibits the activation of antigen-specific T cells by binding to FGL-1, Wang et al. showed that the high expression of FGL-1 was predictive of poor prognosis and was possibly involved in attenuating the therapy effect of anti-PD-1 mAbs; this was related to tumor invasion and metastasis (40), and thus, it was concluded that LAG-3 expression might be associated with anti-PD-1 therapy. The triple blockade (achieved *via* genetic ablation or by using blocking antibodies) of PD-1/CTLA-4/LAG-3 exhibits better and improved effectiveness than the double blockade of PD-1/LAG-3 or PD-1/CTLA-4 in metastatic ovarian cancer. The former strategy can help improve antitumor immunity and inhibit ovarian cancer metastasis; however, the dose, efficacy, and tolerance of patients warrant further exploration (44). As for patients with stage I–IIIB NSCLC with lymph node metastasis, the high expression of LAG-3 is associated with enhanced OS and DSS (disease-specific survival), which can also be considered a predictive factor of NSCLC lymph node metastasis, and LAG-3^+^ tumor-infiltrating lymphocytes can be used to significantly improve disease-specific survival (45); nevertheless, the mechanism through which LAG-3 exerts a negative effect on the immune response helps in improving OS, and disease-specific survival warrants further investigation. LAG-3 is a newly developed immune checkpoint that elicits a potent antitumor immune response and is used as monotherapy or in combination with other therapies. Several clinical studies are underway to investigate the utilization of LAG-3 in tumor metastasis diagnosis and prognosis evaluation, and further studies are warranted to explore the efficacy of and tolerance to LAG-3-targeting mAbs.

### TIGIT and CD155

#### Structure and Expression

TIGIT was first discovered in 2009, and this is a receptor with an IgV domain, a transmembrane domain and an immune receptor tyrosine-based domain (4). The ligand of TIGIT, CD155 (also called PVR), belongs to the nectin-like family, but the conserved amino acids and domain structure are similar to the immunoglobulin superfamily (46). TIGIT and its ligand CD155 are the immune checkpoints expressed on the surface of immune cells; TIGIT is mainly expressed on CD4^+^ T cells, CD8^+^ T cells, NK cells, and Treg cells (47); CD155 is mainly expressed on HM1345^+^ melanoma cells, macrophages, myeloid cells, DCs, and tumor-related infiltration lymphocytes (48).

#### The Immune Mechanism Related to Tumor Metastasis

TIGIT exerts its inhibitory effects on T cells and NK cells by undergoing ligation with CD155 to competitively inhibit the expression of DNAM-1 (CD226), a receptor on the surface of T cells and NK cells that binds to the tumors. Bronte et al. have shown that CD155 is strongly associated with tumor lung metastasis (48), and when CD155 binds to CD96, ITIM (involved in a negative signal transduction pathway) can inhibit the activation of NK cells (49). Currently, studies mainly focus on the TIGIT ligand CD155 and less on TIGIT itself. CD155 is possibly associated with immunosurveillance by establishing interactions with DNAM-1 to inhibit T cell and NK cell-associated cytotoxic effects, which may induce tumor metastasis; however, more *in vitro* experiments are necessary to verify these findings.

#### The Clinical Research Progress Related to Tumor Metastasis

Ailin et al. have demonstrated that because of the resistance to anti-PD-1 treatment in metastatic melanoma, targeting of CD155 offers a novel strategy to promote the function of PD-1^+^CD8^+^ T cells, with improved response to anti-PD-1 therapy (50). The expression of CD155 is associated with breast cancer tumor size, lymph node metastasis, and TNM stage, and boosts the proliferation and metastasis of breast cancer cells by regulating tumor-infiltrating lymphocytes. CD155 is a promising prognostic factor in breast cancer (51). The overexpression of CD155 improves osteosarcoma lung metastasis, and anti-CD155 mAbs can be used to remarkably attenuate lung metastasis by inhibiting the expression of FAK and pFAK, which are associated with cancer cell invasion and metastasis (52). Owing to the high correlation between CD155 and tumor metastasis, the efficacy of bispecific anti-CD3 and anti-CD155 (CD155Bi-Ab) antibodies, with ATC-specific cytotoxic activity, has been tested for the suppression of prostate cancer progression (53). Therefore, CD155 has garnered attention for the development of targeted therapy, and several preclinical trials are in progress; however, there is insufficient clinical evidence to prove its therapeutic effect in humans.

### TIM-3

#### The Structure and Expression

TIM-3 shares a common structure with other TIM family members, which is characterized by five tyrosine residues in the cytoplasmic domain. Regarding its intracellular signaling mechanism, it is known that Tyr256/263 interacts with HLA-B-associated transcript 3(BAT3) and the tyrosine kinase FYN. TIM-3 is a receptor expressed on the surface of CD4^+^ T cells, CD8^+^ T cells, NK cells, DCs, and monocytes (54).

#### The Immune Mechanism Related to Tumor Metastasis

Huang et al. (55) suggested that TIM-3 could be considered to inhibit the development of type-I immunity and to induce peripheral immune tolerance. Solinas et al. (56) proved the suppressive role of TIM-3 in the activation of T cells, which can cause CD8^+^ T cell exhaustion by antagonizing TCR signals. Xiao et al. (57) have demonstrated that TIM-3 is associated with nasopharyngeal carcinoma metastasis *via* the SMAD7–SMAD2–SNAIL1 signaling pathway. The level of SMAD may regulate the expression of SNAIL, which mediates TGF-β-induced epithelial–mesenchymal transition (EMT) and is related to tumor metastasis. SMAD3/4 promotes the transcription of SNAIL and contributes to EMT in skin-related tumors. SMAD2 knockdown effectively increases this effect of SMAD3/4, and inducing the transcription of SMAD7 may prevent TGF-β-induced EMT of tumor cells; thus, SAMD7 and SAMD2 may play a role in EMT to regulate tumor metastasis (58). It has been proven that TIM-3 and PD-1 can be used in synergy, and the double blockade of TIM-3/PD-1 can help amplify T cell activation and restore antitumor immunity (59). Recently, TIM-3 has been found to be negatively associated with tumor metastasis, such as CRC metastasis (5). Considering that TIM-3 is a co-regulator that can simultaneously boost or inhibit the immune response in different types of tumors, the upregulation of TIM3 in immune responses is considered to be related to the increase in IFN-γ. Accordingly, it has been shown that when blocking TIM3, the role of IFN-γ in antitumor immunity is also inhibited (60, 61). The different roles of TIM-3 in different tumors and its participation in various mechanisms warrant further explorations.

#### The Clinical Research Progress Related to Tumor Metastasis

Liu et al. have demonstrated that the high expression of TIM-3 is associated with head and neck squamous cell carcinoma lymph node metastasis and that the mAb targeting TIM-3 can be used to restore the function of CD4^+^TIM-3^+^ and CD8^+^TIM-3^+^ T cells to inhibit tumor growth and metastasis (62). In cases of metastatic NSCLC, exosomal TIM-3 and its ligand galectin-9 are highly associated with tumor size, stage, and distant metastasis, and exosomal TIM-3 is especially correlated with lymph node metastasis (63). Furthermore, the high expression of TIM-3 in metastatic NSCLC helps develop resistance to nivolumab (an anti-PD-1 mAb), and the combination of nivolumab and an anti-TIM-3 mAb may be considered a therapeutic strategy for metastatic NSCLC (64). However, the previously reported findings are based on the high expression of TIM-3 that helps improve tumor metastasis; initially, the role of TIM-3 in metastatic CRC was reportedly the opposite, as the level of TIM-3 in paracancerous tissue was found to be low, and the downregulation of TIM-3 was always associated with lymph node metastasis, distant metastasis (stages III–IV) and tumor cell infiltration or invasion (5, 61). The low expression of TIM-3 is predictive of a poor prognosis of metastatic prostate cancer (65). More evidence is necessary to prove the role of TIM-3 in different tumors, especially in colorectal cancer. Two anti-TIM-3 mAbs, MGB453 (Novartis) and TSR-022 (TESARO), are under phase I/II clinical trials; the results are not published yet; the efficacy of and tolerance to combination immune therapy using anti-TIM-3 and anti-PD-1 mAbs are unknown, but research on TIM-3 has shown considerable potential in tumor metastasis treatment.

### SIGLEC-15

#### Structure and Expression

SIGLEC-15 is a single gene family member with the sialic acid-binding immunoglobulin-type lectin structure, which may help bind with Sialyl-Tn antigen (66). SIGLEC-15 is expressed on the surface of myeloid cells, and the mRNA of SIGLEC-15 is upregulated in various cancers like bladder, kidney, lung and liver cancers, colon cancer, endometrioid cancer, and thyroid cancer (67).

#### The Immune Mechanism Related to Tumor Metastasis

SIGLEC-15 exerts a synergistic effect with PD-1/PD-L1, being able to deliver a negative signal by binding to a presumptive receptor on T cells, which can suppress the activation of antigen-specific T cells and restore antitumor immunity in the TME. hS15-hIg and mS15-mIg (fusion proteins of SIGLEC-15) can inhibit the activation of T cells in both humans and mice (66). This immune checkpoint has been demonstrated to be exceedingly innovative but has been the focus of few reports; its role in tumor metastasis remains unclear.

#### The Clinical Research Progress Related to Tumor Metastasis

It has recently been discovered that SIGLEC-15 is expressed in 90% of NSCLC cases, and a phase I clinical trial is underway (NCT03665285) (67). However, although SIGLEC-15 seems to be a novel immune checkpoint, its effects on other tumors and mechanisms in tumor metastasis remain unclear, and more preclinical and clinical trials are necessary to validate its effects.

### IDO-1

#### Structure and Expression

IDO-1 is a rate-limiting enzyme and can convert tryptophan into kynurenines, which is expressed on the surface of APC and epithelial-derived tumor cells (68, 69).

#### The Immune Mechanism Related to Tumor Metastasis

Regarding the relationship between IDO-1 and immune response, which may be exerted *via* pathways like NF-κB, JAK/STAT, PKC, and TGF-β (68, 69), the depletion of Trp is implicated in elevated tRNA levels of T cells to anergic T cells *via* activation of the GCN2 and mTOR signaling pathways, and the expression of Kyn is related to T cell necrosis, which further enables the conversion of CD4^+^ T cells into Tregs (70). Data have demonstrated synergy through the combined use of anti-PD-1 and anti-IDO-1 mAbs (ORR increases from 10 to 51%). Nonetheless, studies should be conducted to further elucidate the immune mechanisms of IDO-1 and may help optimize the curative effect and restrain drug resistance.

#### The Clinical Research Progress Related to Tumor Metastasis

A growing body of evidence suggests that anti-IDO-1mAbs are not effective when administered as a single agent. The anti-IDO1 mAb D-1-MT works as a pathway inhibitor, and the ORR associated with D-1-MT monotherapy of solid tumors or metastatic solid tumors is approximately 10–18%, while in combination with anti-PD-1 or anti-CTLA-4 mAbs, the ORR increases to 51% (70). IDO-1 plays a crucial role in tumor metastasis in many cancer types, such as gastric cancer, and the increased expression of IDO-1 and expression of the core gene COL12A1 synergistically improve cancer cell invasion and metastasis *via* the MAPK transduction pathway (71). For patients with brain metastasis attributable to lung cancer, the expression of IDO-1 is markedly increased (72), and a phase II clinical trial (NCT02085070) has shown a 33% response rate after the administration of pembrolizumab (anti-PD-1 mAb) in patients with lung cancer brain metastasis, thus supporting that the higher expression of IDO-1 is associated with better response to anti-PD-1 treatment (6). In combination with IDO-1, which can promote NSCLC lymph node metastasis and invasion, p53 suppresses the pathway of IDO-1, thereby indicating that the increased expression of p53 can inhibit NSCLC metastasis. IDO-1 is not expressed in metastatic renal cell carcinoma, but the expression of IDO-1 on tumor epithelial cells can be used to predict better PFS and therapeutic response to nivolumab. A few phase I/II trials (NCT 02178722) reported thus far have evaluated the efficacy and safety of the IDO-1 inhibitor epacadostat plus pembrolizumab (73). Concurrently, phase I/II clinical trials (NCT 01604889) were conducted to explore the safety of and tolerance to epacadostat plus ipilimumab in metastatic melanoma, concluding that the dose of epacadostat that exerts effects and can be well tolerated is ≥25 mg BID, while for patients with advanced metastatic melanoma it is ≤50 mg BID (74). A phase I clinical trial (NCT 02048709) was conducted to assess the curative effect and irAEs of the IDO-1 inhibitor navoximod in recurrent advanced solid tumors, but it failed to prove its effect in metastatic tumors, and thus, further investigation is warranted (75). Although many experimental and theoretical studies indicate that IDO-1-based treatment strategies have progressed in recent years, especially in combination with other ICIs, such as pembrolizumab and ipilimumab, more clinical trials should explore the efficacy and safety of D-1-MT and epacadostat in the treatment of several other metastatic tumors, regardless of their use as monotherapies or combination therapies.

### B7 Family (VISTA, VTCN1, CD276)

#### Structure and Expression

VISTA is a type I transmembrane protein with 309 amino acid, which consists of 136 amino acid single IgV extracellular domain, 23 amino acid stalk domain, 21 amino acid domain, and 97 amino acid cytoplasmic domain (76). Higher expression of VISTA can be discovered on the surface of T cells, activated Treg cells, myeloid cells, and mature APC cells (77).

VTCN1 is a kind of transmembrane protein with 282 amino acids, consisting of a transmembrane domain, a two amino acid intracellular domain and seven N-glycosylation site extracellular domain (78), which is a type of minority immune checkpoint that is expressed on the surface of tumor cells like serum ovarian cancer and breast cancer (79).

The extracellular structure of CD276 consists of two IgC and variable (IgV) domains; it is inherently a type I transmembrane protein (80). Similar to VTCN1, CD276 is mainly expressed on tumor cells, such as lung cancer, renal cell carcinoma, breast cancer, endometrial cancer, and ovarian cancer cells (81).

#### The Immune Mechanism Related to Tumor Metastasis

VISTA serves as a receptor for the inhibition of CD4^+^ T cells (82) by binding to its ligand VIG-3 (83) and subsequently increases the threshold of T cell activation by inhibiting the relay of TCR signals (84). Herrera et al. found that VISTA can be used in synergy with PD-1 and that both can be used simultaneously to inhibit the activation of T cells (82).

VTCN1 was identified as a negative regulator of T cell activation and cytokine production, especially inhibiting CD4^+^T cell differentiation to T_H_1 or T_H_17 cells, and thus causing a transient increase in the population of Tregs *via* the establishment of a potential mechanistic link with IL-6 and IL-10 (79, 85). VTCN1 is intricately associated with various tumor metastases, which can be attributed to the inhibition of T cells in the tumor microenvironment.

Initially, CD276 was recognized as an immune stimulatory molecule, but the immune suppressive function of this molecule has also been reported, indicating that CD276 can be used in synergy with PD-1 and CTLA-4. In addition to inhibiting the secretion of cytokines such as IFN-γ and TNF-α, CD276 can also inhibit the proliferation and activation of T cells. Moreover, the decreased expression of CD276 leads to a reduction in the expression of metastasis-related proteins such as MMP-2, STAT3, and IL-8 (86).

#### The Clinical Research Progress Related to Tumor Metastasis

Gao et al. have shown that the expression of PD-L1 and VISTA increases following treatment with ipilimumab in metastatic prostate cancer, suggesting that VISTA may be relevant to prostate cancer metastasis (21). VISTA expression is positively correlated with advanced stage, lymph node metastasis, and poor prognosis in ovarian cancer, but not with the 5-year OS (87). In metastatic melanoma, the downregulation of VISTA, PTEN, and HLA is evidently associated with anti-PD-1 mAb resistance, and it has been discovered that pharmacological inhibition of the VISTA signal pathway may be considered to synergistically enhance the efficacy of anti-PD-1 therapy (88). Considering the clinical significance of VISTA, research efforts have been engaged toward the elucidation of the relationship between VISTA and tumor metastasis; however, drug research on VISTA is far from realization.

The overexpression of VTCN1 and Ki-67 in the nuclear membrane may be considered a novel and independent marker of poor prognosis in patients with metastatic pleural carcinoma (89). For pancreatic cancer, the median OS was 9.90 ± 0.58 months (95% CI, 8.76–11.04) months, and the expression of VTCN1 was predictive of distant metastasis and poor prognosis with decreased OS (3.80 ± 0.84 months, 95% CI, 2.16–5.44) (90). Upregulation of VTCN1 expression in lung carcinoma is strongly associated with lymph node metastasis and the TNM stage, and VTCN1 can facilitate the growth and proliferation of tumor cells by upregulating the expression of Bax and caspase3/8 and by downregulating that of Bcl-2 and cyclin_D1 (91). The downregulation of PI3K/AKT/mTOR leads to the decreased expression of VTCN1, which inhibits the metastasis of colorectal carcinoma (92), as reviewed above. The PI3K axis is related to PD-1, and it is reasonable to investigate the interaction between the former axis and PD-1/VTCN1. Recently, it was discovered that VTCN1 is related to the metastasis of melanoma, prostate cancer, renal cell carcinoma, breast cancer, and ovarian cancer (93, 94). Furthermore, substantial research progress has been made on VTCN1 targeting drugs, but the results are unsatisfactory. Owing to the potential correlation between VTCN1 and PD-1, this immune checkpoint has application potential in the development of antitumor therapies.

Seaman et al. have reported that the anti-CD276 IgG1 antibody m276 (human) is anergic, but anti-CD276 mAb enoblituzumab is activated with an enhanced Fc, and m276-PBD (mouse) can increase the OS in a mouse model (5 months, 88% treatment group, no tumors detectable). High expression of CD276 is observed in lung and liver metastases of colon cancer, as well as in breast cancer metastases to the lung (81), and Zhang et al. have reported that patients with lymphatic metastasis of NSCLC, advanced TNM staging, and poor prognosis usually present increased CD276 expression (95). Dong et al. concluded that the pharmacological inhibition of CD276 exerts remarkable effects when CD276 is located on the cell membrane, and the bispecific antibody anti-CD3 × anti-CD276 has been shown to exert significant antitumor activity and to inhibit lung cancer metastasis. Yu et al. have reported that CD276 is overexpressed in lung adenocarcinoma and is associated with lymph node and distant metastases, and CD276 expression can promote EMT of lung adenocarcinoma cells and facilitate tumor infiltration and metastasis (96). High expression of CD276 is strongly associated with prostate cancer metastasis, poor prognosis, and high mortality, which is regulated by androgen and TGF-β signaling pathways (97). Karlsen et al. have reported that the combination of an anti-CD276 mAb with certain small-molecule inhibitors, such as the BRAF inhibitor vemurafenib, the MEK inhibitor binimetinib, the mTOR inhibitor everolimus, and the AKT inhibitor triciribidine, is effective in the treatment of metastatic melanoma, increasing the sensitivity of tumor cells to treatment and suppressing melanoma cell metastasis, proliferation, and invasion (98). A similar strategy may be adopted in breast cancer; low expression of CD276 enhances cancer cell sensitivity to paclitaxel treatment and AKT/mTOR inhibitors (99). These results indicate that CD276 may exert better effects when administered with other molecular inhibitors. Using the SSIGN score, the expression of CD276 may be evaluated and considered a novel biomarker to predict the short-term survival of patients with metastatic clear renal cell carcinoma (100). Additionally, CD276^+^ fibroblasts can be used to promote tumor progression and metastasis (101). CD276 can also help promote pancreatic carcinoma metastasis and infiltration by activating the TLR4–NF-κB signaling pathway, *via* upregulated expression of IL-8 and VEGF (102). Research on CD276 has progressed mainly regarding the lung cancer subtype; however, more clinical trials and animal models that allow the application of anti-CD276 mAbs are warranted to evaluate its efficacy and safety.

### CD70

#### Structure and Expression

CD70, a member of the TNF family, is a type II transmembrane glycoprotein and composed of 193 amino acids, with over 20 membrane-bound and secreted protein ligands (103). CD70 is much more regulated and expressed on the surface of activated T, B cells, thymic medulla epithelial cells, DCs, and APC cells (104).

#### The Immune Mechanism Related to Tumor Metastasis

CD70 exerts its functions *via* the CASPASE, JNK, and NIK/IKK signaling pathways to establish interactions with CD27. CD70 can aid the creation of an immunosuppressive TME and inhibit the activation of NK cells, reduce the expression of MHC proteins, and attenuate T cell activation. Furthermore, CD70 can activate Tregs and lead to the apoptosis of lymphocytes, as well as inhibit T cell inflammatory response, especially that of CD4^+^ T cells (104). SGN-CD70A is a widely studied anti-CD70 mAb, and a few irAEs occur when it is used; however, the involved immune mechanisms are unknown and may be associated with the inhibition of the immune system.

#### The Clinical Research Progress Related to Tumor Metastasis

It has been shown that CD70 expression is associated with the development of multiple cancers, such as lymphoma, renal cell carcinoma, nasopharynx cancer, and metastatic tissues. CD70 reaches up to 100% expression, which can also induce Tregs in NSCLC. There are three mAbs targeting CD70, namely SGN-CD70A, AMG172, and ARGX110. SGN-CD70A is considered for the treatment of metastatic renal cell carcinoma and recurrent NHL (Non-Hodgkin lymphoma). Clinical trials using SGN-CD70A are underway to define the maximum tolerance dosage (104, 105), and the main reported irAEs are thrombocytopenia, fatigue, anemia, and peripheral edema (106). Liu et al. have reported that CD70^+^ breast cancer stem cells preferentially metastasize to the lungs. CD70 can promote the proliferation and differentiation of tumor cells in metastatic tissues, indicating that CD70 may be considered a novel predictive factor for metastasis (107). However, data accumulated thus far on CD70 are controversial. The function of CD70 shows an opposite trend in metastatic melanoma, in which the expression of CD70 is reduced, and CD70^+^ cancer cell populations are remarkably decreased in metastatic sites, indicating that CD70 may inhibit the ability of tumor metastasis. Moreover, the absence of CD70 always results in the creation of a suppressive tumor immune microenvironment *via* MAPK pathways, RhoE, and cytoskeletal modulation (108). CD70 serves as an inhibitory checkpoint for the immune system and plays a significant role in tumor metastasis; nevertheless, its role is controversial because it has been described to promote the metastasis of breast cancer but also the inhibition of melanoma and thus, more evidence is necessary to further detail its dual role. Regarding the application of AMG172 and ARGX110, few studies have been published. We further anticipate the study of irAEs associated with the above-mentioned mAbs and the underlying mechanisms.

### CD47

#### Structure and Expression

CD47 is a kind of glycoprotein and composed of 5-time transmembrane spanning domain, single extracellular V-set immunoglobulin, and short cytoplasmic region (109). CD47 is regularly expressed on all the normal cells and overexpressed on tumor cells (110).

#### The Immune Mechanism Related to Tumor Metastasis

CD47 demonstrates its functions *via* SIRPα to inhibit macrophage phagocytosis, help tumor cells escape immune surveillance, and inhibit the elicitation of both the innate and adaptive immune responses. Some mAbs available target CD47, namely TTI-621 and Hu5F9; the former fuses to the N domain of SIRPα to suppress the inhibition of CD47, and the latter is a human antibody that inhibits the interaction between CD47 and SIRPα and can boost macrophage phagocytosis (110). The most notable characteristic of CD47 is its interaction with macrophages; further studies on anti-CD47 mAb may help explore the role of macrophages in tumor metastasis and the effect of enhancing macrophage phagocytosis for the inhibition of tumor metastasis.

#### Clinical Research Progress Related to Tumor Metastasis

CD47 is predictive of the prognosis of NHL, bladder, and breast cancers. The melanoma immune microenvironment in primary and metastatic sites can be influenced by CD47 expression, which is associated with melanoma metastasis *via* the inhibition of macrophages (111). Kulthida et al. have shown that CD47 promotes the growth and metastasis of cholangiocarcinoma (CCA); anti-CD47 mAb can inhibit the proliferation of CCA cells in primary and metastatic sites by inducing macrophage phagocytosis, and the interaction between CD47 and SIRPα is critical in this process; thus, targeting CD47 and SIRPα may be considered a novel precise therapeutic approach for preventing intrahepatic metastasis of CCA (112). It is evident that high expression of CD47 promotes tumor invasion and metastasis through Cdc42, and the level of CD47 is associated with tumor stage and lymph node and distant metastasis, especially in NSCLC; therefore, CD47 can be used as a biomarker for the prognosis of NSCLC. Furthermore, its downstream regulator Cdc42 induces tumor metastasis, and thus, both CD47 and Cdc42 may be considered potential therapeutic targets to control NSCLC (113). Hu et al. have reported that the overexpression of CD47 in CRC is associated with poor prognosis and metastasis and that the CD47–ENO1 axis may provide a promising insight into the development of targeted therapies for CRC (114). The anti-CD47 antibody Hu5F9-G4 is being tested in 62 patients with solid malignancies and lymphoma in an ongoing phase I clinical trial (NCT02216409) that aims to investigate its priming dose and maintenance dose. Hu5F9-G4 can induce macrophage phagocytosis and thus inhibits tumor metastasis. Another antibody, AMMS4, can suppress the growth and metastasis of solid tumor cells by improving macrophage infiltration (115). There is a more effective bispecific antibody, rituximab–anti-CD47, that can help regulate the innate immune response, inhibit the interaction between CD47 and SIRPα, and increase macrophage infiltration and may demonstrate a better therapeutic effect and antitumor function (116). Many new forms of such therapy are currently in clinical development, including the blockade of CD47 and combination therapies. So far, CD47 is the only immune checkpoint that can be considered to inhibit macrophage phagocytosis, which renders anti-CD47 therapies different from others that target immune checkpoints to inhibit tumor metastasis.

## Immune-Activating Checkpoints

### TNFSF and TNFRSF

#### Structure and Expression

TNFSF14 [LIGHT is homologous to lymphotoxins and engages the herpes virus entry mediator (HVEM) and the Lymphotoxin-β receptor] is a member of the TNFSF family; CD16, IL-2, and IL-15 engage in the activation of NK cells, which promotes the expression of TNFSF14 on the surface of activated T cells, NK cells, and immature DCs (117).

TNFRSF4 (OX40, CD134) is essentially a 50 kDa type I transmembrane protein and characterized with four cysteine-rich domains. TNFRSF4 is expressed on CD4^+^ and CD8^+^ T cells, NK, NKT cells, and neutrophils (118, 119).

The glycoprotein TNFRSF9 (CD137,4-1BB) was first discovered in 1989 and shares a common structure with other TNFRSF family members, which is composed of 255 amino acids, 2 N-linked glycosylation, 169 amino acid extracellular domain, and 27 amino acid transmembrane domain (120). TNFRSF9 is known as an immune-stimulant molecule expressed on the surface of T cells, NK cells, DCs, Tregs, NKT cells, neutrophils, monocytes, and eosinophils (121). TNFRSF18 (GITR) is also a member of TNFRSF family and a 26 kDa type I transmembrane protein with 241 amino acids (122). TNFRSF18 is expressed on epithelial cells, T cells, DCs, and the CD4^+^FOXP3^+^ Treg cells in tumors (123).

#### The Immune Mechanism Related to Tumor Metastasis

TNFSF14 can help regulate T-cell co-stimulation *via* binding to HVEM and LTBR. Additionally, it can increase CD8^+^ T cell infiltration in tumors, normalize tumor vessels, and induce the production of cytokines such as IFN-γ, TNF-α, and MIG, which may inhibit tumor metastasis (124).

TNFRSF4 is an immunostimulatory molecule that activates T cells and memory T cells to boost the proliferation of CD4^+^ and CD8^+^ T cells and stimulates the differentiation of CD4^+^ T cells into T_H_1 and T_H_2 cells through the NF-κB and NFAT signaling pathways (125). TNFRSF4 mainly acts by enhancing the capacity of T cells, which can inhibit tumor metastasis by significantly stimulating adaptive immunity and triggering a positive change in the immune tumor microenvironment.

The ligand of TNFRSF9 is 4-1BBL, which promotes the activation and proliferation of CD4^+^ and CD8^+^ T cells, the cytotoxic effect of CD8^+^T cells, and the differentiation of effector memory CD8^+^ T cells *via* TRAF1/2, NF-κB, AP-1, JNK, and MAPK signals to enhance the APCC cytotoxic effect of NK cells (121). TNFRSF9 plays a specific role in tumor metastasis, which consists in stimulating the immune system, but is simultaneously associated with tumor metastasis; the reason for the occurrence of this phenomenon remains unclear.

Besides the regulation of co-stimulatory molecules on ATCs, TNFRSF18 exerts its functions *via* NF-κB signaling pathways, with the engagement of two molecules downstream of p53, namely p21 and PUMA, to restore the function of tumor-infiltrating lymphocytes (123). Knockout of TNFRSF18 is associated with changes in the immune tumor microenvironment and cytokine secretion.

#### The Clinical Research Progress Related to Tumor Metastasis

The normalization of tumor vessels can be induced by TNFSF14, which subsequently inhibits the metastasis of melanoma and lung cancer. Regarding the antitumor function of TNFSF14, considerable progress has been achieved through studies using animal models with the aim to explore the therapeutic effect of the anti-TNFSF14 mAb LIGHT-VTR in combination with an anti-PD-1 mAb; favorable results have been reported in the reduction of lung cancer metastasis (124). However, the study by Brunetti shows completely opposite results and suggests that high serum levels of TNFSF14 are significantly related to bone metastasis of NSCLC, with the activation of osteocytes *via* the RANK–RANKL–OPG signaling pathway (126). Therefore, the role of TNFSF14 in NSCLC metastasis is controversial and warrants further evidence; the mAb targeting TNFSF14 is under clinical trial, and the tolerance to TNFSF14 and combination effects are unclear.

The clinical trials conducted to assess the effect of anti-TNFRSF4 mouse mAb 9B12 show unfavorable results; 40% of solid tumors are reportedly advanced with at least one-site metastasis. Presently, its antitumor effect is explored in combination with 9B12 and anti-PD-1 and anti-CTLA-4 mAbs as well as other antitumor treatments in melanoma and prostate and breast cancers (127, 128). Approximately three-fourths of ovarian cancer cases exhibit metastasis to the peritoneal cavity, and the TNFRSF4 fusion protein mCTH-ANXA5 is used in the treatment of metastatic ovarian cancer. Additionally, the combination of anti-CD73, anti-TNFRSF4, and mCTH-ANXA5 mAbs can be used to significantly improve the survival rate (12–24 days for all murine objects) and to reduce the burden of metastatic ovarian cancer by enhancing the function of cytotoxic T cells (129). Similarly, the combination of TNFSF4 (OX40L) fusion protein and poxvirus-based cancer vaccine (MVA-Twist-TRICOM) can also be used effectively to inhibit lung metastasis of breast cancer by increasing the infiltration of CD4^+^CD8^+^ T cells and the production of IFN-γ and TNF-α (130). The agonist anti-TNFRSF4 antibody is used to inhibit metastasis of metastatic cutaneous squamous cell carcinoma (mSCC) by significantly inhibiting Tregs and by improving the infiltration of tumor-associated CD4^+^ T cells (131). In metastatic melanoma, the low expression of OX40L is an indicator of poor prognosis for all patients with metastatic melanoma, especially for patients with stage III–IV disease, which is associated with decreased lymphocyte infiltration (132). Presently, certain antibodies targeting TNFRSF4 are being evaluated in clinical trials, such as 9B12, BMS 986178, PF-04518600, and MEDI6469. 9B12 is being used in an ongoing phase I clinical trial, and we anticipate further exploration of its effect and patient tolerance (133). Currently, there are an increasing number of studies focusing on TNFRSF4, and the above-mentioned trial has identified TNFRSF4 as a promising immune checkpoint that can lead to the elicitation of an antitumor immune response for the inhibition of metastasis.

Owing to its positive effect on the immune system, the antibody targeting TNFRSF9 can lead to the activation of T cells and enhance the function of CD8^+^ T cells to suppress the invasion and metastasis of tumors. The serum level of TNFRSF9-L is elevated in patients with metastatic breast cancer and is associated with bone metastasis *via* increases in the infiltration of macrophages and facilitation of its differentiation into osteoclasts. However, although TNFRSF9 exerts positive immunomodulatory function on immune cells like macrophages, it can also improve tumor metastasis (134), and thus, further studies are warranted to explore its molecular basis. Two agonist antibodies, namely urelumab (BMS-663613) and utomilumab (PF-05082566), can be used in synergy with anti-CD20 and anti-PD-1 mAbs and are under study in clinical trials for the confirmation of their therapeutic effect on metastatic melanoma and advanced solid tumors, especially NSCLC and renal cell carcinoma (121). Certain individuals develop severe AEs when the administered dose of urelumab exceeds 1 mg/kg every 3 weeks, and the well-tolerated dose is 0.1 mg/kg every 3 weeks, which is acceptable for exploring the therapeutic effect of urelumab, regardless of its administration as monotherapy or combination therapy on advanced solid tumors; however, its safety profile in metastatic tumors is unclear (7). A phase I clinical trial has demonstrated that utomilumab is well tolerated by patients with advanced tumors; its irAEs are <10%, and the overall ORR is 3.8% in solid tumors; however, the therapeutic effect of combining utomilumab with other ICIs such as pembrolizumab remains to be explored, along with the application potential of single-agent umilumab in melanoma and NSCLC, which are cancer types that are resistant to anti-PD-1/PD-L1 therapy (135). In the case of TNFRSF9, antitumor drug research is rapidly developing, and the safety of both antibodies has been assessed in advanced solid tumors but not in metastatic tumors.

Animal studies on TNFRSF18 have shown that this checkpoint is related with the development of bladder carcinoma, breast cancer, SCLC, and melanoma; the knockout of TNFRSF18 leads to the accumulation of tumor cells, and the high expression of BRCA/HNSC is a favorable prognostic indicator. Owing to the methylation of CpG islands in advanced multiple myeloma, tumors progress with decreased expression of TNFRSF18 (123, 136). In metastatic renal cell carcinoma, the combination of sunitinib with an α-GITR agonist can be used to effectively improve the tumor immune microenvironment and to activate the cytotoxicity of CD8^+^ T cells and NK cells to prevent liver metastasis (8). The treatment using intravenous tumor-primed CD4^+^ T cells with intraperitoneal administration of an α-GITR mAb can activate CD8^+^ T cells and increase the secretion of cytokines such as TNF-α, IL-4, and IL-5, which may inhibit tumor metastasis; the study showing these findings (NCT02583165, NCT02628574) is currently part of an ongoing phase I clinical trial on breast cancer and melanoma. However, its potential applicability to tumors that have metastasized warrants investigation (137).

### CD28

#### Structure and Expression

CD28 initiates a cascade of intracellular events like the production of cytokines and T cell differentiation with extracellular immunoglobulin-like domain, which belongs to the co-stimulatory subfamily (138). CD28 can be expressed on various kinds of cells like CD4^+^ and CD8^+^ T cells, as well as bone marrow stroma cells, neutrophils, eosinophils (139).

#### The Immune Mechanism Related to Tumor Metastasis

CD28 is a type of co-stimulatory TCR that can function independently and can be used in synergy with PD-1 or CTLA-4. CD28 can function positively and negatively (establishes interactions with CTLA-4) simultaneously; however, Esensten et al. have shown that its positive effect is markedly greater than its negative effect and that CD28 provides the secondary messenger to activate T cells by establishing interactions with B7-1 (CD80) and B7-2 (CD86). Additionally, CD28 can promote the function of effector T cells and Tregs *via* TGF-β and Lck signals to inhibit TCRs (138).

#### The Clinical Research Progress Related to Tumor Metastasis

Yan et al. have reported that CD28 is irrelevant to metastatic NSCLC prognosis; however, the serum level of CD28 is higher in patients with breast cancer and has been identified as a novel prognostic indicator (140, 141). Based on the results reported by Marco, the upregulated expression of CD28 correlated with a better benefit in patients with malignant melanoma treated with ipilimumab, indicating that CD28 can serve as a prognostic factor in melanoma (142). More research efforts have been engaged in elucidating the interaction between CD28 and PD-1 or CTLA-4 and in ascertaining whether it is possible to enhance the therapeutic effect of anti-PD-1 or anti-CTLA-4 by modulating CD28 expression.

## Discussion

This review summarizes the latest research progress reported over the last 5 years on 17 immune checkpoints to clearly highlight aspects as to their expression **(** **Table 1** **),** immune mechanism **(** **Table 2** **)**, relationship with tumor metastasis, and clinical application **(** **Table 3** **)**. Through the information presented herein, we aimed to provide insights into various potential approaches that could be adopted for developing tumor immune therapeutic options in the future and discussed the recent research progress on the clinical response involving immune checkpoints.

**Table 1:** Immune checkpoints structure and expression.

Immune checkpoints	Structure and expression
PD-1	① 288 amino acid and a single IgV extracellular domain ② on the surface of activated T cells, B cells, NKT cells, NK cells, and macrophages
CTLA-4	① a 36-amino acid cytoplasmic tail ② on the surface of activated T cells and Treg cells as a receptor
LAG-3	① D1–D4 four immunoglobulin-like domains ② on the surface of activated CD4^+^ T cells, CD8^+^ T cells, NK cells, and Treg cells
TIGIT	① the IgV domain, transmembrane domain and an immune receptor tyrosine-based domain ② on the surface of CD4^+^ T cells, CD8^+^ T cells, NK cells, and Treg cells
TIM-3	① 5 tyrosines in the cytoplasmic domain ② on the surface of CD4^+^ T cells, CD8^+^ T cells, NK cells, DCs, and monocytes
SIGLEC-15	① the sialic acid-binding immunoglobulin-type lectin structure ② on the surface of myeloid cells, and in various types of cancers
IDO-1	on the surface of APC and epithelial-derived tumor cells
VISTA (B7-H5)	① 309 amino acids include 136 amino acid single IgV extracellular domain 23 amino acid stalk domain 21 amino acid domain 97 amino acid cytoplasmic domain ② on the surface of T cells, activated Treg cells, myeloid cells, and mature APC cells
VTCN1 (B7-H4)	① 282 amino acids include a transmembrane domain a 2 amino acids intracellular domain 7 N-glycosylation sites extracellular domain ② on the surface of tumor cells
CD276 (B7-H3)	① two IgC and variable (IgV) domains ② on the surface of tumor cells
CD70	① 193 amino acids, with over 20 membrane bound and secreted protein ligands ② on the surface of activated T, B cells, thymic medulla epithelial cells, DC cells, and APC cells
CD47	① 5-time transmembrane spanning domain, single extracellular V-set immunoglobulin,short cytoplasmic region ② on all the normal cells and overexpressed on tumor cells
TNFSF14 (LIGHT)	① homologous to lymphotoxins and engages the herpes virus entry mediator (HVEM) and the Lymphotoxin-*β* receptor ② on the surface of activated T cells, NK cells and immature DCs
TNFRSF4	① 4 cysteine-rich domains ② on CD4+ and CD8+ T cells, NK, NKT cells and neutrophils
TNFRSF9	① 255 amino acid include 2 N-linked glycosylation 169 amino acid extracellular domain 27 amino acid transmembrane domain ② on the surface of T cells, NK cells, DCs, Treg, NKT cells, neutrophils, monocytes, and eosinophils
TNFRSF18 (GITR)	① 241 amino acids ② on epithelial cells, T cells, DCs, and the Treg cells in tumors
CD28	① extracellular immunoglobulin-like domain ② on CD4^+^, CD8^+^ T cells, bone marrow stroma cells, neutrophils and eosinophils

**Table 2:** The immune mechanism related to tumor metastasis.

Immune Checkpoint	Tumor-Metastasis-Related Immune Response
**Immune inhibitors**	
PD-1 and PDL-1/2	① inhibit proximal T cell receptor and CD28 ② attenuate activated T cells co-stimulation ③ inhibit the activation of peripheral activated T cells ④ PTEN can boost the above three ⑤ Akt can promote the expression of PDL-1
CTLA-4	inhibits self-reactive T cells by binding with B7 to competitive inhibit CD28 co-stimulation
LAG-3	① competitively binds with MHC-II ② negative regulation for T cell expansion with boosting T cell exhaustion ③ activates DCs
TIGIT and CD155	① has a synergistic effect to interact with CD155(TIGIT ligand) and inhibit T cells and NK cells ② TIGIT binds with CD155 and competitively inhibits DNAM-1 ③ ITIM (a negative signal transduction pathway CD155 binds with CD96) can inhibit the activation of NK cells
TIM-3	① inhibits type-I immunity and peripheral immune tolerance ② inhibits the components of TCR signals, stops the activation of T cells, and boosts the exhaustion of CD8^+^ T cell ③ TIM-3 can work with PD-1
SIGLEC-15	① delivers a negative signal by binding with a presumptive receptor on T cells and suppresses the activation of antigen-specific T cells ② restores the role of antitumor immunity in TME ③ hS15-hIg and mS15-mIg can be used to inhibit T cell activation ④ SIGLEC-15 can work with PD-1
IDO-1	① The deletion of Trp makes tRNA of T cells out of control ② Activating the signal transduction pathways of GCN2 and mTOR makes T cell anergic signals ③ The expression of Kyn is related with T cells necrosis, rendering CD4^+^ T cells into Tregs ④ IDO-1 can work with PD-1
VISTA (B7-H5)	① inhibits CD4+ T cells by binding with its ligand VIG-3 ② increases the threshold of T cells activation *via* inhibiting TCR signals ③ VISTA can work with PD-1
VTCN1 (B7-H4)	① inhibits the proliferation of CD4+T cells and its differentiation toward Th1, Th17 cells ② makes the transient increase of Tregs ③ inhibits the secretion of cytokines
CD276 (B7-H3)	① inhibits the proliferation and activation of T cells and the secretion of some cytokines like IFN-*γ* and TNF-α ② The decreased expression of CD276 will lead to the tumor-metastasis-related protein MMP-2, STAT3, IL-8 get reduced signals ③ CD276 can work with PD-1 and CTLA-4
CD70	① renders the TME into the suppressive environment, the activation of NK cells will be inhibited, the expression of MHC protein can be reduced, T cells can be anergic ② activates Tregs and lead to the apoptosis of lymphocytes, as well as inhibits T cells inflammatory response, especially for CD4^+^T cells signals ③ CD70 works *via* CASPASE, JNK and NIK/IKK signal pathways to interact with CD27
CD47	① CD47 works *via* SIRPα to inhibit the phagocytosis of macrophages ② Represses innate and adaptive immune ③ Helps tumor cells to escape from immune surveillance
**Immune activators**	
TNFSF14 (LIGHT)	① regulates T cells co-stimulation *via* binding with HVEM and LTBR and boost the infiltration of CD8^+^T cells into tumors ② normalizes the tumor vessels ③ makes some cytokines increased, such as IFN-*γ*, TNF-α, MIG
TNFRSF4	TNFRSF4 mainly promotes the capacity of T cells ① activates T cells and memory T cells ② boosts the proliferation of CD4^+^ and CD8^+^ T cells, to accelerate CD4^+^ T cells to differentiate into Th1 and Th2 cells *via* NK-*κ*B and NFAT signal transduction pathways
TNFRSF9	① accelerates the activation and proliferation of CD4^+^ and CD8^+^ T cells ② accelerates the cytotoxic effect of TCR and CD8^+^ T cells ③ accelerates the differentiation of effector memory CD8^+^ T cells ④ enhances the APCC cytotoxic effect of NK cells
TNFRSF18 (GITR)	① regulates the role of co-stimulatory molecules in the activation process of effector T cells ② restores the function of tumor infiltration lymphocytes
CD28	① provides the second messenger for TCR to facilitate T cells activation ② promotes the function of effector T cells and Tregs ③ CD28 can work with PD-1 and CTLA-4

**Table 3:** The progress of clinical research related to tumor metastasis.

Immune Checkpoint	Related Tumors	Monoclonal Antibody
**Immune inhibitors**		
PD-1 and PDL-1/2	Prostate cancer	PD-1: Nivolumab, Pembrolizumab, Camrelizumab, Cemiplimab
	Melanoma	
	NSCLC	
	Breast cancer	
	Nasopharyngeal carcinoma	PDL-1: Atezolizumab, Avelumab (MSB0010718c phase I and II clinical trials) Durvalumab
	Gastric cancer	
	MCC(Merkel cell carcinoma)	
	Urothelial carcinoma	
CTLA-4	Melanoma	Ipilimumab
	Colorectal cancer	
	Advanced renal cell carcinoma	
	Colon cancer	
	NSCLC	
LAG-3	Ovarian cancer	No mAb
	NSCLC	Being synergistic with anti-PD-1 and anti-CTLA-4
TIGIT	Melanoma	Bispecific antibody anti-CD3 and anti CD155 (CD155Bi-Ab)
	Breast cancer	
	Osteosarcoma	
	Prostate cancer	
TIM-3	HNSCC	① MGB453 (Novartis)
	NSCLC	② TSR-022 (TESARO)
	Colorectal cancer	③ Both under phase I/II clinical trials
	Prostate cancer	
SIGLEC-15	NSCLC	Ongoing phase I clinical trial
IDO-1	Gastric cancer	① D-1-MT
	Lung cancer	② Epacadostat (is ongoing phase I/II trial)
	Melanoma	③ Navoximod (is ongoing phase I trial)
		Being synergistic with Anti-PD-1 and anti-CTLA-4
		Exerts by combining with p53
VISTA (B7-H5)	Prostate cancer	No mAb
	Ovarian cancer	Being synergistic with anti-PD-1
	Melanoma	
VTCN1 (B7-H4)	Pleural carcinoma	No mAb
	Pancreatic cancer	
	Lung carcinoma	
	Colorectal carcinoma	
	Melanoma	
	Prostate cancer	
	Renal cell carcinoma	
	Breast cancer	
	Ovarian cancer	
CD276 (B7-H3)	Colon cancer	① m276 (human antibody)
	Breast cancer	② m276-PBD (mouse antibody)
	NSCLC	
	Prostate cancer	
	Melanoma	
	Renal cell carcinoma	
	Pancreatic carcinoma	
CD70	Lymphoma	① SGN-CD70A (is ongoing phase I trial)
	NHL	② AMG172
	Renal cell carcinoma	③ ARGX110
	Nasopharynx cancer	
	Melanoma	
CD47	NHL	① Hu5F9-G4 (is ongoing phase I trial)
	Bladder cancer	② AMMS4
	Breast cancer	
	HNSCC	
	Melanoma	
	CCA	
	NSCLC	
	Colorectal cancer	
**Immune activators**		
TNFSF14 (LIGHT)	Melanoma	LIGHT-VTR
	Lung cancer	
TNFRSF4	Melanoma	① 9B12 (is ongoing phase I trial)
	Prostate cancer	② BMS 986178
	Breast cancer	③ PF-04518600
	Ovarian cancers	④ MEDI6469
	CSCC	Being synergistic with anti-PD-1 and anti-CTLA-4
TNFRSF9	Breast cancer	① Urelumab (BMS-663613)
	Melanoma	② Utomilumab (PF-05082566 is ongoing phase I trial)
	NSCLC	
	Renal cell carcinoma	Being synergistic with anti-PD-1
TNFRSF18(GITR)	Bladder carcinoma	*α*-GITR (NCT02583165, NCT02628574, are ongoing phase I trial)
	Breast cancer	
	SCLC	
	Melanoma	
	Renal cell carcinoma	
CD28	NSCLC	Being synergistic with anti-PD-1 and anti-CTLA-4
	Breast cancer	
	Melanoma	

MCC, Merkel cell carcinoma; NSCLC, non-small cell lung cancer; SCLC, small cell lung cancer; CCA, cholangiocarcinoma; NHL, non-Hodgkin’s lymphoma; HNSCC, head and neck squamous cell carcinoma; CSCC, cutaneous squamous cell carcinoma.

The application of anti-PD-1 antibodies is a milestone in tumor immune therapy, and certain immune checkpoints can be used in synergy with PD-1, as reviewed above. TIM-3 and VISTA can be used in synergy with PD-1 to inhibit the activation of T cells, restore their function, and enhance antitumor immunity. SIGLEC-15 is a novel immune checkpoint that acts synergistically with PD-1. The dual blockade of PD-1 and CTLA-4, conducted to simultaneously inhibit CD276, can help in inhibiting the activation of T cells and cytokine secretion, thereby also downregulating LAG-3 expression and improving antitumor immunity to achieve inhibition of ovarian cancer metastasis. The combination approach based on the inhibition of PD-1 and activation of TNFSF14 demonstrates a favorable effect on the suppression of lung tumor metastasis. CD28 is an independent immune checkpoint and can also be considered a ligand of PD-1 or CTLA-4, as it may interact with CTLA-4 and enhance the curative effect of ipilimumab. Data indicate an improved outcome of the blockade of both IDO-1 and PD-1, which can increase the ORR from 10 to 51% in metastatic solid tumors.

Ipilimumab (targeting CTLA-4) has been widely used and associated with severe irAEs, which may be attributed to the wide-spectrum immune suppression; hence, novel strategies to counter this drawback are urgently needed.

In addition to PD-1/PD-L1 and CTLA-4, other immune checkpoints have also been reported to function as novel prognostic indicators. For example, the expression of LAG-3 and VTCN-1 is indicative of lung cancer metastasis; TIM-3, CD47, and OX-40L are predictive of the prognosis of metastatic tumors such as prostate cancer, NHL, and melanoma; CD276 is associated with the survival of patients with renal cell carcinoma; and IDO-1 may be associated with the therapeutic effect of nivolumab. Therefore, these immune checkpoints may serve as prognostic indicators and possess diagnostic value for metastatic tumors.

Owing to ICIs’ negative regulation of tumor metastasis, in theory, their usage is considered for the treatment of early stage tumors to inhibit their development and reduce the probability of tumor metastasis. A phase II clinical trial using NADIM (143) indicated that neoadjuvant immunotherapy (nivolumab) combined with chemotherapy for patients with early stage NSCLC is associated with a favorable outcome (ORR, 76%; DCR, 100%). A phase III clinical trial by Bristol-Myers Squibb (New York, USA) (Checkmate-816) also showed similar results; compared to the group administered with chemotherapy alone (pCR, 2.2%), the group treated with immunotherapy combined with chemotherapy showed a pCR of up to 24%. Moreover, the efficacy of other PD-1 or PD-L1 inhibitors, like pembrolizumab (PEARIS, NCT02504372), durvalumab (BR31, NCT02273375), and atezolizumab (IMPOWER010, NCT02486718), in treating early stage lung cancer is being evaluated in ongoing clinical trials. Nevertheless, there is not enough evidence to prove the effect of ICIs in preventing tumor metastasis, and a long-term follow-up of patients that receive ICIs at the early stage of the disease is needed to accurately evaluate the efficacy of ICI in preventing tumor metastasis. Furthermore, for patients with metastatic cancer, only a few mAbs can be used as therapy. For instance, camrelizumab (anti-PD-1 mAb) can be used for patients with recurrent/metastatic nasopharyngeal carcinoma, showing an ORR of 34.1%. The combination of D-1-MT (an anti-IDO-1 mAb) with anti-PD-1 or anti-CTLA-4 mAbs in the treatment of metastatic solid tumors increases the ORR up to 51%.

Many experimental and theoretical studies have shown that various mAb combinations, such as rituximab–anti-CD47, can help improve macrophage infiltration and regulate the innate immune response. The blockade of PD-1/CTLA-4/LAG-3 can efficiently inhibit ovarian cancer metastasis, thus showing a favorable outcome in reducing the risk of tumor metastasis. The combination of LIGHT-VTR and anti-PD-1 therapy can effectively inhibit lung cancer metastasis in animal models, but there is no report on the clinical combination of immune inhibitors and activators. One reason is that there are no immune checkpoint activators yet available for clinical use; another is that the dual activation may result in an extremely high incidence of irAEs, which leads to immune overactivation prior to immune reactivation. Regarding fatal irAEs, compared to the 32-day median time from symptom onset to death associated with ICI monotherapy, that of ICI combination therapy is only 14 days, indicating that the latter strategy may lead to more severe irAEs (144). Thus, it is unclear whether serious and fatal adverse effects occur before the antitumor effects when the combination of immune inhibitors and activators is used. Nonetheless, data also indicate that patients with irAEs have a better response rate, progression-free survival, and OS than those without irAEs (13). Further studies are warranted to explore the effect of the combination of immune inhibitors and activators as well as the therapeutic effect and AEs of different combinations in different tumors. Several studies show considerable advantages of and potential for implementing immune checkpoint combination therapies in tumor metastasis, which represents the subsequent steps of treatment development. NF-κB may become the intersection point of immune inhibitors and activators. Regarding immune inhibitors, IDO-1 regulates the immune response *via* NF-κB; CD276 can help promote pancreatic carcinoma metastasis and infiltration by activating the TLR4–NF-κB signaling pathway, *via* upregulated expression of IL-8 and VEGF. As for immune activators, TNFRSF (including TNFRSF4/9/18) highlights the role of NF-κB in T cell activation and differentiation. Thus, it is possible to consider NF-κB as a co-regulator in immune inhibition and activation.

Although significant progress has been achieved in oncology, researchers continue to explore targeted drugs for CD28, VISTA, and VTCN1; however, no significant progress has been reported thus far. The development of mAbs targeting CD70, CD276, and TIGIT has achieved little success and is being investigated in preclinical trials. Meanwhile, there are a few reports available on the role of immune checkpoints in neurological tumors.

Taken together, our knowledge of these immune checkpoints has increased substantially, and more theoretical, preclinical, and clinical studies are underway. The approaches necessary to maximize the curative effect and to minimize the AEs of immune checkpoint-targeting therapies should be explored in the future. The diverse strategies and the changes in the immune microenvironment may influence various aspects of immune therapy. Additionally, similar immune mechanisms may be considered to lay the foundation for combination therapy.

## Author Contributions

X-YT and A-PS are mainly responsible for paper writing and typesetting. Y-LX, K-FZ, Y-JL, and X-GS are mainly responsible for references collecting and table-form making. J-BZ and TJ are mainly responsible for paper review. All authors contributed to the article and approved the submitted version.

## Funding

Natural Science Basic Research Project of Shaanxi Province, No.2016JM8087. National Natural Science Foundation of China, No. 82002421.

## Conflict of Interest

The authors declare that the research was conducted in the absence of any commercial or financial relationships that could be construed as a potential conflict of interest.
